# Disseminated varicella with systemic implications in a renal transplant recipient^☆^

**DOI:** 10.1016/j.abd.2022.10.013

**Published:** 2023-07-05

**Authors:** Ingrid Rocha Meireles Holanda, Marina Oliveira Dias, Rebecca Perez de Amorim, Aline Lutz Garcia, Ricardo Augusto Monteiro de Barros Almeida, Silvio Alencar Marques

**Affiliations:** a Department of Dermatology, Universidade Estadual Paulista, Faculty of Medicine, Botucatu, SP, Brazil; b Department of Infectology, Dermatology, Diagnostic Imaging and Radiotherapy, Universidade Estadual Paulista, Faculty of Medicine, Botucatu, SP, Brazil

Dear Editor,

Varicella is an infectious and contagious disease caused by the varicella-zoster virus (VZV) transmitted by aerosols or direct contact with active skin lesions. In immunocompetent children, it is usually a self-limited benign disease, but more severe, potentially fatal forms can occur in children over 12 years of age and in immunosuppressed patients.

## Case report

A 22-year-old male patient referred pruritic skin lesions for four days, initially seen on the abdomen with dissemination to the rest of the skin. The lesions were preceded by epigastric pain radiating to the mesogastrium and hypogastrium, of moderate intensity and showing a continuous pattern, associated with hyporexia, nausea and vomiting. He denied fever, respiratory symptoms, or other complaints. He had been submitted to renal transplantation six months before due to bladder malformation and since then had been receiving immunosuppressive therapy with tacrolimus 3 mg/day, mycophenolate mofetil 2 g/day and prednisone 5 mg/day. He denied a history of varicella and had an incomplete vaccination card, with no record of vaccination against varicella.

**Figure 1:**
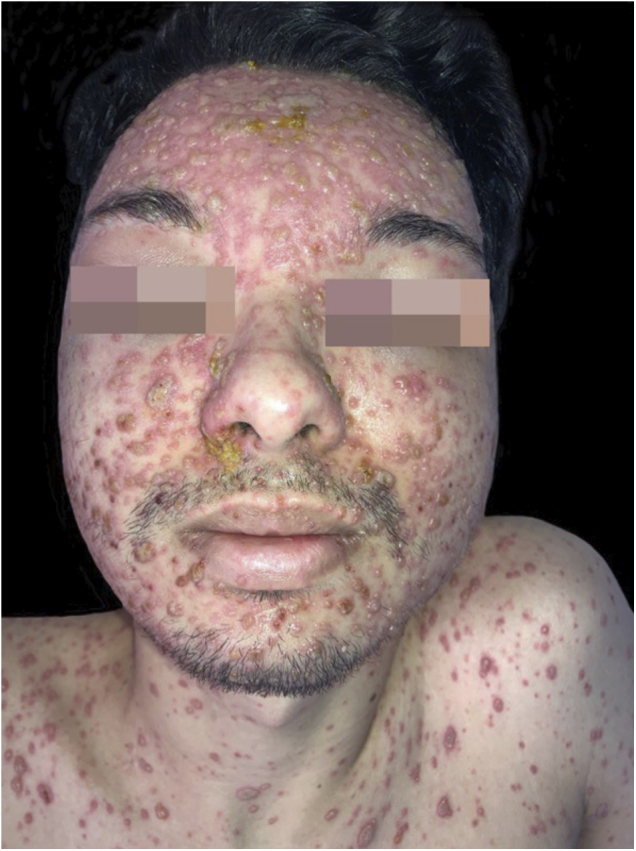
Varicella in a renal transplant patient: multiple vesico-bullous lesions, mostly monomorphic, on an erythematous base, disseminated over the face, some covered by meliceric crusts

**Figure 2:**
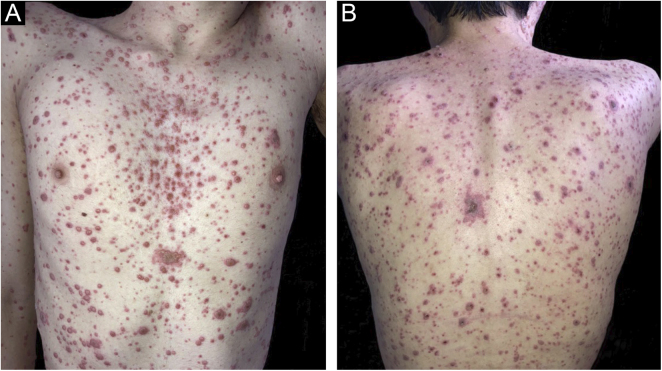
(A) Varicella in renal transplant patient: isolated and confluent lesions, disseminated over the trunk. (B) Progression to a necrohemorrhagic pattern

On physical examination, numerous disseminated vesicles and bullae were observed on an erythematous base, particularly on the trunk and face (Figure 1, Figure 2), including the oral and genital mucosa.

Upon admission, laboratory tests, cytological examination and biopsy of an intact vesicle were requested. In addition, treatment with intravenous acyclovir was immediately started at a dose of 10 mg/kg/ every 8 h during in-hospital isolation.

**Figure 3:**
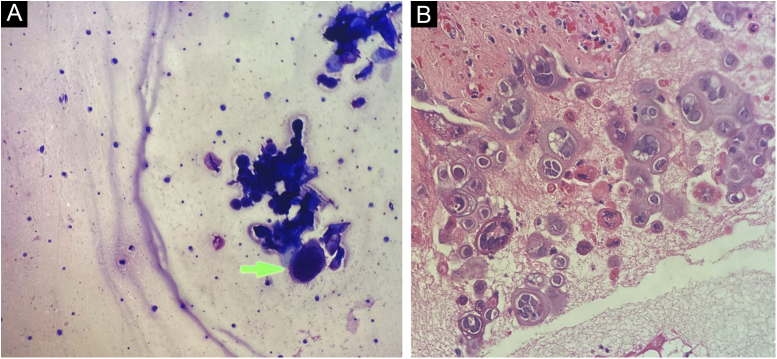
(A) Multinucleated cells with nuclear enlargement and ground-glass appearance (Tzanck cytology). (B) Enlarged multinucleated epithelial cells, with molded nuclei and “ground-glass” chromatin (Hematoxylin & eosin ×40)

Cytological examination of Tzanck smears showed multinucleated squamous cells, with large nuclei, and ground-glass appearance, which are cytological alterations compatible with the cytopathic effect caused by herpes virus (Fig. 3A).

The anatomopathological examination showed a bullous lesion with intraepidermal cleavage, containing enlarged, cytopathic, multinucleated epithelial cells with molded nuclei, and “ground glass” chromatin (Fig. 3B). Further investigation with immunohistochemistry for HSV-1 and -2 antibodies, and Grocott-Gomori and Ziehl-Neelsen staining were negative.

**Figure 4:**
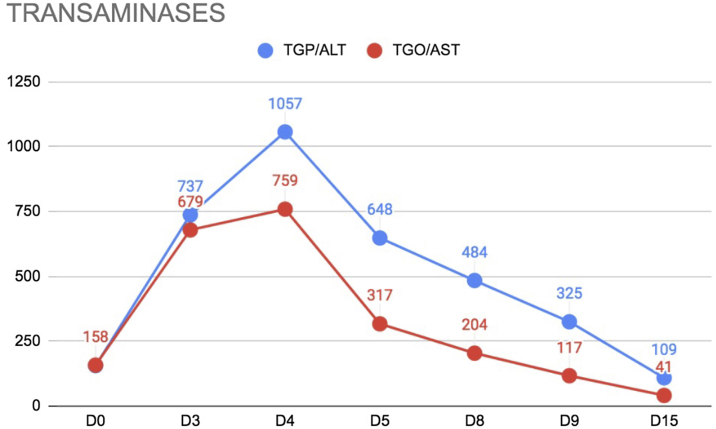
Curve of liver enzymes during the period of the disease and treatment with intravenous acyclovir. D0, First day of hospitalization; D15, Day of hospital discharge

Laboratory tests showed progressive elevation of the liver (Fig. 4) and canalicular enzymes. RT-PCR for COVID-19 was performed during hospitalization, although the patient had no respiratory symptoms, with a positive result. Computed tomography (CT) of the chest was performed, showing small foci of opacities and scattered nodular consolidation throughout the lung parenchyma, predominantly in the periphery of the left lung, some with a ground-glass halo, alterations suggestive of viral infection, more compatible with VZV infection than with SARS-CoV 2. An abdominal CT was performed, showing homogeneous hepatosplenomegaly.

During hospitalization, skin lesions increased in number, some showing a necrohemorrhagic pattern, associated with liver and lung complications. One week after starting treatment, dermatological and systemic improvement was observed, and after two weeks, the patient had all the lesions in the crusting phase and progressed to healing, with pain improvement, and progressive decrease in liver and canalicular enzymes.

He was discharged from the hospital under transition from acyclovir (D15) to oral administration, at a dose of 800 mg, five times a day until reaching 21 days of treatment. When reassessed, the patient was fully recovered, in good general condition, and showed healed lesions.

This case report describes the clinical case of a renal transplant patient, receiving immunosuppressive therapy, with no previous history of varicella, who had not been vaccinated against the disease and who had not been submitted to a pre-transplant vaccination card update.

The varicella-zoster virus is a pathogenic human alpha-herpesvirus that causes varicella as a primary infection. It is a highly contagious disease, most commonly seen in children under ten years of age.^1^

Its transmission occurs in susceptible hosts, through contact with nasopharyngeal secretion aerosols from an infected individual, or through direct skin contact with vesicular fluid from active skin lesions and has an incubation period of 10 to 21 days. The period of infectivity is considered to last from 48 hours before the onset of the rash until the skin lesions have fully progressed to the crusting phase.^2^

In immunocompetent children, it is usually a self-limited benign disease, progressing to cure within seven to ten days. However, more severe forms can occur in people over 12 years of age and in immunosuppressed patients. In these, the infection may present with a longer period of fever, a greater number of lesions, prolonged duration, and risk of complications.^3^, ^4^

The most common complication is infection of the lesions by *Staphylococcus aureus* or *Streptococcus pyogenes*, ranging from pyoderma to cellulitis, toxic shock syndrome – staphylococcal or streptococcal, and even necrotizing fasciitis.^5^

Moreover, neurological complications, pneumonia, and hepatitis are not uncommon, either.^6^, ^7^ The most frequently described neurological changes are cerebellar ataxia and encephalitis.^6^

Pneumonia frequently occurs in immunosuppressed patients, and is the main cause of death. Symptoms begin soon after the appearance of the rash and include coughing, dyspnea, and even hemoptysis. CT shows nodular and diffuse interstitial infiltrates Before antiviral therapy and intensive care support, case mortality reached 30%, with rapid progression to death. Currently, mortality is less than 10%.^6^

In immunosuppressed patients, liver damage is caused by a direct viral cytopathic effect on hepatocytes. Clinical symptoms include abdominal pain of varying intensity, and the skin lesions may precede, coincide with, or appear after hepatitis, which may delay the specific diagnosis.^6^, ^7^

The diagnosis of a VZV infection is usually clinical; however, further investigation is recommended, including serology, culture, biopsy, and Tzanck testing.

In adult patients, individuals with complications, and/or immunocompromised ones, treatment with antivirals is mandatory. Oral therapy is used in immunocompetent individuals without evidence of complications, for five to seven days. In immunocompromised patients, intravenous acyclovir is indicated, at a dosage of 10 mg/kg/IV every 8 h, until there is significant symptoms improvement and no new lesions occur. Transition to oral therapy is recommended and should be continued until the lesions progress to the crusting and scarring phase.^8^

Infection prevention is of the utmost importance for the growing population of solid-organ transplant recipients and immunosuppressed ones for different reasons. Immunization with the varicella vaccine is recommended for patients at risk, at least four weeks before transplantation or immunosuppressive therapy.^9^, ^10^ It is important that these patients receive two doses of varicella vaccine, if possible, with a minimum interval of four weeks between doses for subjects aged ≥ 13 years and a minimum interval of three months for subjects aged 1 to 12 years.^9^, ^10^

## Financial support

None declared.

## Authors' contributions

Ingrid Rocha Meireles Holanda: Design and planning of the study; drafting and editing of the manuscript; collection, analysis, and interpretation of data; effective participation in research orientation; intellectual participation in the propaedeutic and/or therapeutic conduct of the studied cases; critical review of the literature; critical review of the manuscript; approval of the final version of the manuscript.

Marina Oliveira Dias: Design and planning of the study; drafting and editing of the manuscript; collection, analysis, and interpretation of data; intellectual participation in the propaedeutic and/or therapeutic conduct of the studied cases; critical review of the literature; critical review of the manuscript.

Rebecca Perez de Amorim: Drafting and editing of the manuscript; collection, analysis, and interpretation of data; intellectual participation in the propaedeutic and/or therapeutic conduct of the studied cases; critical review of the literature; critical review of the manuscript.

Aline Lutz Garcia: Drafting and editing of the manuscript; collection, analysis, and interpretation of data; intellectual participation in the propaedeutic and/or therapeutic conduct of the studied cases; critical review of the literature; critical review of the manuscript.

Ricardo Augusto Monteiro de Barros Almeida: Drafting and editing of the manuscript; collection, analysis, and interpretation of data; intellectual participation in the propaedeutic and/or therapeutic conduct of the studied cases; critical review of the literature; critical review of the manuscript.

Silvio Alencar Marques: Design and planning of the study; drafting and editing of the manuscript; effective participation in research orientation; intellectual participation in the propaedeutic and/or therapeutic conduct of the studied cases; critical review of the literature; critical review of the manuscript; approval of the final version of the manuscript.

## Conflicts of interest

None declared.
